# The characteristics of tissue microbiota in different anatomical locations and different tissue types of the colorectum in patients with colorectal cancer

**DOI:** 10.1128/msystems.00198-25

**Published:** 2025-05-27

**Authors:** Lei Liu, Jianguo Shi, Hui Wang, Hansong Du, Jia Yang, Kai Wei, Zhuohui Zhou, Moli Li, Shuai Huang, Lifang Zhan, Guolong Li, Yongling Lv, Hexiao Shen, Wei Cai

**Affiliations:** 1 Department of Gastrointestinal Surgery and Intestinal Microenvironment Treatment Center, The Central Hospital of Wuhan, Tongji Medical College, Huazhong University of Science and Technology 12443, Wuhan, Hubei, China; 2 Hubei Provincial Engineering Research Center of Intestinal Microecological Diagnostics, Therapeutics, and Clinical Translation 675140 , Wuhan, Hubei, China; 3 School of Life Sciences, Hubei University 12563, Wuhan, Hubei, China; Argonne National Laboratory, Lemont, Illinois, USA

**Keywords:** gut microbiota, colorectal cancer, intratissue bacteria, right-sided colon, microbiome, functional analysis

## Abstract

The gut microbiota is intricately associated with the onset and progression of colorectal cancer (CRC), leading to significant interest in developing prevention and treatment strategies that leverage gut microbiota. In this study, we collected 57 samples from 19 CRC patients, comprising cancerous tissue, paracancerous tissue, and normal mucosa. Utilizing metagenomic sequencing and bioinformatics analysis, we identified differences in the microbiomes and their functional characteristics across the various tissue types. The results indicated that species such as *Alistipes putredinis* were predominantly found in normal tissues, while *Pseudomonas putida* was enriched in paracancerous tissue, and *Malassezia restricta* was prevalent in cancerous tissues. Furthermore, the microbial functions exhibited variability among the different tissue types. Random forest analysis suggested that *Moraxella osloensis* may be implicated in the onset and progression of colorectal cancer. We also classified the patients into three subgroups based on the anatomical location of the colorectum: right-sided colon, left-sided colon, and rectum. The subgroup analysis revealed that the microbiota enriched in normal mucosa and paracancerous tissue varied across different anatomical sites. These findings not only elucidate the characteristics of the microbiomes in the normal mucosa, paracancerous tissue, and cancerous tissues of CRC patients, thereby providing new potential targets for clinical diagnosis and treatment, but also contribute to the existing microbiome data pertinent to CRC research.

**IMPORTANCE:**

This study provides crucial insights into the relationship between gut microbiota and colorectal cancer (CRC) by analyzing microbial communities in different tissue types and anatomical locations of CRC patients. We identified distinct microbial signatures, such as *Alistipes putredinis* in normal tissues and *Malassezia restricta* in cancerous tissues, indicating location-specific microbiomes with unique functional attributes. These findings suggest potential new biomarkers or therapeutic targets for CRC. The observed microbiota variations among right-sided colon, left-sided colon, and rectum cancers underscore the heterogeneity of CRC, pointing toward more personalized treatment strategies. By enhancing our understanding of the microbiome’s role in CRC, this research paves the way for innovative diagnostic tools and targeted therapies tailored to individual patient profiles. This work is essential for advancing clinical approaches to CRC management.

## INTRODUCTION

According to data from the World Health Organization’s CANCER TODAY initiative, colorectal cancer (CRC) has emerged as the second leading cause of cancer-related mortality worldwide, with an estimated 1.926 million new cases and 904,000 deaths annually (1). The regions exhibiting the highest incidence and mortality rates of CRC predominantly include Europe, Oceania, and the Americas, which may be associated with Western dietary patterns (2). A growing body of research has established a significant correlation between the occurrence of cancer and the gut microbiome. The composition of the host’s gut microbiome is influenced by both environmental factors and host genetics, resulting in an ecological imbalance that is linked to carcinogenic effects (3, 4). Among various malignancies, CRC demonstrates a direct interaction with the gut microbiome (5, 6). Currently, the relationship between the gut microbiome and the onset, progression, and treatment of CRC is garnering increasing attention from academia and clinicians, and related research holds substantial significance for the early screening and efficacy assessment of CRC.

The gut microbiome constitutes a significant element of the gut microecology and is intricately associated with gut health. The gut microbiome is predominantly located in the feces within the intestinal lumen, and microbial communities are also found in both normal colorectal tissue and colorectal tumor tissue. Dysbiosis of the gut microbiota has been implicated in the development of CRC. Analyses of fecal microbiome metagenomic sequencing data from both healthy individuals and CRC patients have shown a significant reduction in the levels of *Roseburia* spp. in CRC patients (7). Several studies comparing tissue and fecal microbiota between CRC patients and healthy controls have also reported a significant decrease in the levels of *Eubacterium* and *Roseburia* spp. in the patient cohort (8, 9). Furthermore, experimental evidence supports the anti-colorectal cancer activity of the intestinal bacterium *Eubacterium callanderi* (10). Additionally, specific bacteria such as *Fusobacterium nucleatum* and *Parvimonas micra* have been associated with the carcinogenesis of CRC (11, 12). The proliferation of certain pathogenic bacteria, along with their metabolites and virulence factors, resulting from dysbiosis of the gut microbiota, can facilitate the occurrence and progression of CRC (12). Interventions aimed at modulating the gut microbiota have the potential to enhance the host’s anti-tumor responses and mitigate intestinal toxic side effects (12). A systematic review of CRC patients who underwent surgical treatment assessed the impact of probiotics on various postoperative indicators, revealing that probiotic administration can significantly reduce inflammation levels, as well as decrease the incidence of postoperative infections and adverse effects, while also modifying the composition of the fecal microbiota and the microbial community within tumor tissue (13). The characteristic microbiota identified in these investigations can modulate inflammation and immune responses through their structural components or metabolites, thereby influencing the incidence and treatment outcomes of CRC. However, due to regional variations across studies, limited sample sizes, and inconsistencies in the delineation of specific tissue sites, a consensus on a characteristic microbiota associated with the incidence or treatment of CRC has yet to be established globally. Consequently, further research is warranted to explore this topic in greater depth.

In this study, 57 samples were collected from 19 CRC patients, including normal mucosa, paracancerous tissue, and cancerous tissue. Analysis based on metagenomic sequencing showed that species such as *Alistipes putredinis* were relatively enriched in normal tissue, species such as *Pseudomonas putida* were relatively enriched in adjacent tissue, and *Malassezia restricta* was relatively enriched in cancer tissue. This finding suggests the presence of disparities in the distribution of microbial communities across diverse tissue samples. Given the anatomical distinctions between the right-sided colon, left-sided colon, and rectum, as well as their clinical implications, exploratory subgroup analyses were conducted based on distinct intestinal sites. The results obtained from this analysis indicated that the species enriched in normal mucosa and paracancerous tissue exhibited variations in accordance with the intestinal site, while no significantly enriched species were found in cancer tissue. These results indicate that the normal mucosa, paracancerous tissue, and cancer tissue of CRC patients possess distinct microbial characteristics, suggesting the presence of microbial specificity across diverse intestinal anatomical sites. These differential microbial communities encompass species with potential protective or pathogenic functions, which may offer novel targets for clinical diagnosis and treatment of CRC. Furthermore, these findings contribute to the enrichment of metagenomic data concerning intestinal microbiota in CRC, thereby facilitating the advancement of scientific research in this field.

## MATERIALS AND METHODS

### Research subjects and sample collection

This study included 19 colorectal cancer (CRC) patients who underwent surgical treatment in the Department of Gastrointestinal Surgery at Wuhan Central Hospital from February to July 2023. All patients had not received radiotherapy or chemotherapy prior to surgery and were categorized into three groups based on the tumor location: right-sided colon cancer, left-sided colon cancer, and rectal cancer. Three distinct types of colorectal tissue samples were obtained from the surgically resected specimens of each patient: normal mucosa (≥ 10 cm away from the tumor focus at the proximal end of the surgical specimen), paracancerous tissue (1–2 cm away from the tumor focus at the proximal end of the surgical specimen), and cancerous tissue (located at the tumor focus) for subsequent metagenomic sequencing analysis. The samples were organized according to tissue type into the normal mucosa group (N_total), paracancerous mucosa group (P_total), and cancer tissue group (C_total). And they were further categorized by anatomical location into the right colon cancer group (N_RC, P_RC, C_RC), left colon cancer group (N_LC, P_LC, C_LC), and rectal cancer group (N_Rectum, P_Rectum, C_Rectum) (Table 1). All samples were immediately stored at −80°C following collection until the DNA extraction process. The detailed information regarding the samples is enumerated in Table S1. In Text S1, we provided a detailed description of the definition of RC, LC, and the methods for sample collection.

**TABLE 1:** Demographic and clinical characteristics of patients^*a*^

Characteristic	RC (*n* = 7)	LC (*n* = 7)	Rectum (*n* = 5)	*P*-value^*b*^
No. male/female	2/5	4/3	3/2	
Age (yrs)	67 ± 11.68	57.29 ± 14.37	71.6 ± 8.11	0.29
BMI	21.63 ± 1.95	21.36 ± 2.99	22.24 ± 1.45	0.71
Tumor diameter (cm), max	3.93 ± 0.89	5.64 ± 3.35	4.74 ± 1.51	0.49
IIA/III B/III C	2/4/1	3/4/0	4/0/1	–
Differentiation				
Well	1	2	1	–
Well to moderate	1	0	1	–
Moderate	3	4	1	–
Moderate to poor	2	1	1	–
Poor	0	0	1	–
Tumor location				–
Cecum	2	0	0	–
Ascending colon	4	0	0	–
Proximal transverse colon	1	0	0	–
Descending colon	0	1	0	–
Sigmoid colon	0	6	0	–

^*a*^ RC, right colon; LC, left colon; BMI, body mass index.

^*b*^ *P*-value: statistical significance calculated using ANOVA for continuous variables and Fisher’s exact test for categorical variables. –, not applicable.

### DNA extraction and quality assessment

Total DNA was extracted using the QIAamp DNA Mini Kit (Qiagen), and each batch of experiments included a negative control to monitor contamination. The concentration and purity of the extracted DNA were assessed using a NanoDrop spectrophotometer, while DNA integrity was verified through agarose gel electrophoresis to ensure compatibility with high-throughput sequencing, and the A260/A280 ratio was maintained between 1.8 and 2.0.

### Metagenomic sequencing

For each sample, we conducted whole-genome metagenomic sequencing using paired-end sequencing on the Illumina NovaSeq 6000 platform, generating approximately 10 Gb of raw data with an average read length of 150 bp to ensure adequate coverage depth. A separate library was prepared for each sample utilizing the TruSeq DNA PCR-Free Library Prep Kit, and library quality was evaluated using the Agilent Bioanalyzer 2100 system.

### Bioinformatics analysis

The raw sequencing data underwent rigorous quality control, employing Trimmomatic v0.39 to remove low-quality reads and adapter sequences, followed by KneadData v0.10.0 to filter out host sequences. Clean reads were assembled into contigs using MEGAHIT (version v1.2.9), discarding any contigs shorter than 500 bp. Prodigal (version V2.6.3) was then utilized to identify coding sequences (CDSs) on the contigs, with CDSs shorter than 100 bp also being removed. CD-HIT (version 4.8.1) was employed to cluster the genes obtained from each sample and eliminate redundancy, applying thresholds of coverage >90% and identity >95%. The TPM (transcripts per million) value for each gene in each sample was calculated using Salmon, while Kraken2 (v 2.1.2) was used for sequence annotation and to determine species abundance, which was subsequently refined using Bracken. Functional annotation was conducted based on the KEGG and GO databases

### Statistical analysis

Statistical analysis was conducted using the R programming language along with its associated packages. For comparisons of continuous variables, the Mann-Whitney U test or the Kruskal-Wallis H test was employed, while categorical variables were analyzed using the chi-square test. A *P*-value of less than 0.05 was deemed statistically significant. Alpha diversity indices, including the Shannon index, and beta diversity assessments, such as the Bray-Curtis distance matrix, were utilized to evaluate differences in microbial community structures among samples. In addition, multidimensional scaling analysis (MDS) and principal coordinate analysis (PCoA) were implemented to visualize the similarities and differences between the samples.

## RESULTS

### Assessment of the quality of metagenomic sequencing data

This study conducted metagenomic sequencing on 57 collected samples, yielding a total of approximately 721.83 Gb of sequencing data (Table S2). The average sequencing depth for each sample was 12.66 Gb, and the average Q30 value, which serves as an indicator of sequencing quality, was 96.54%. These results suggest that both the sequencing depth and quality of the samples were sufficiently high to proceed with subsequent analyses. However, due to the elevated host contamination rates associated with mucosal tissue and the relatively low microbial content, quality control measures were implemented to remove host contamination. Following these procedures, the average amount of bacterial genomic data available for analysis per sample was 94.64 Mb.

### Variations in microbiota composition and functional attributes across distinct types of colorectal tissue

**Fig 1:**
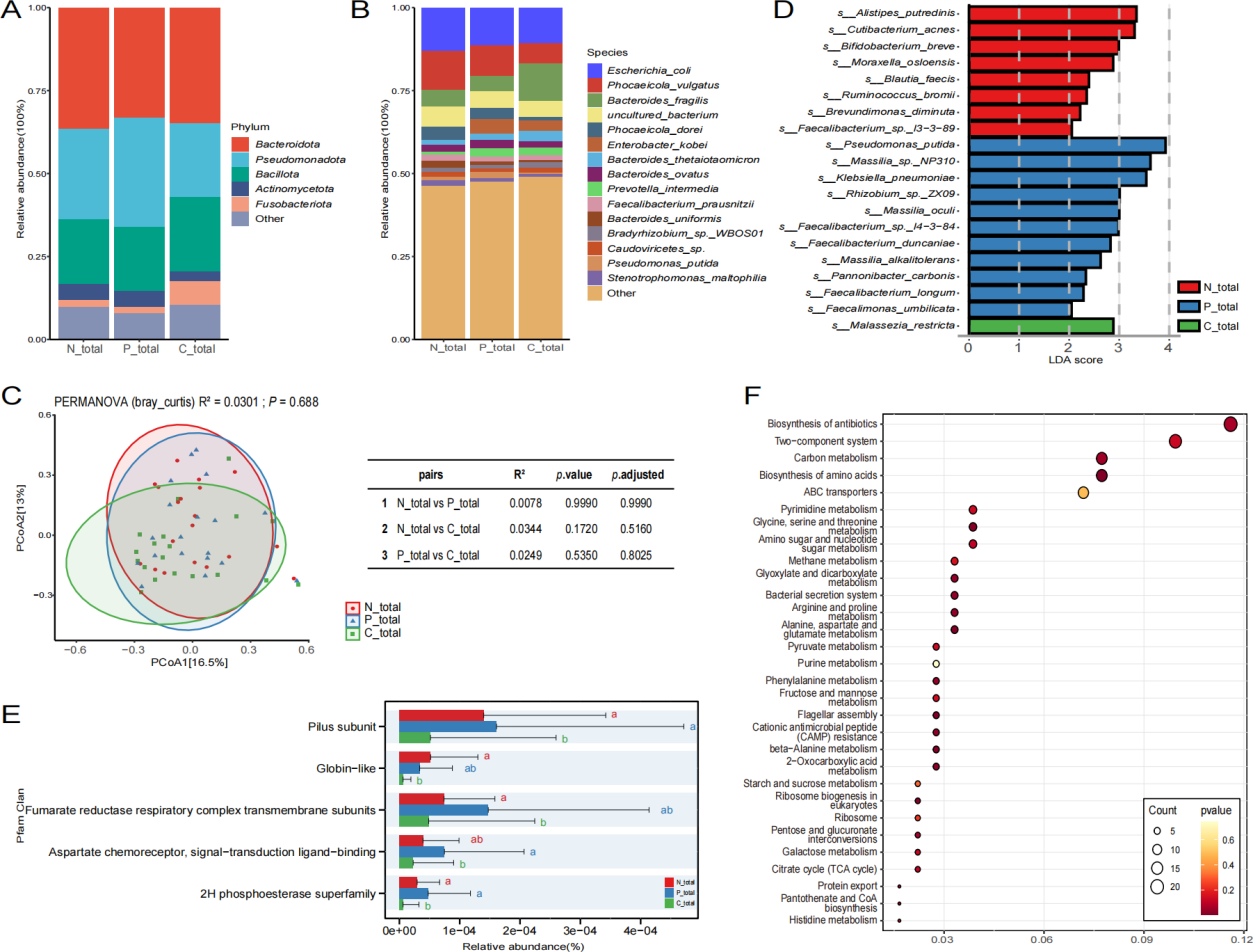
The microbiome analysis results of various types of colorectal tissues are presented as follows. Panels A and B illustrate the species composition at the phylum and species levels using bar graphs. Panel C displays the results of the Bray-Curtis β-diversity analysis, where the shapes of the data points indicate different groups. Panel D features the results of the LEfSe analysis, with the horizontal axis representing the LDA score and the vertical axis denoting the types of microbial species. Panel E shows a bar graph of the Pfam functional difference analysis, in which the horizontal axis represents relative abundance and the vertical axis represents different functional categories; letters that differ between the two groups indicate significant differences, while the absence of differing letters signifies no significant difference. Panel F presents a bubble chart depicting differential KEGG functions, where the horizontal axis denotes the Rich factor—with larger values indicating a greater proportion of genes enriched in the pathway relative to the total number of genes in that pathway—while the vertical axis indicates different functions. The size of the circles corresponds to the count of genes enriched in each function, and the color of the circles represents the *P*-value.

This study classified the 57 collected samples into three distinct categories: normal mucosa (N_total), paracancerous tissue (P_total), and cancerous tissue (C_total). The microbiome data from these categories were analyzed to elucidate variations in their respective microbiomes. As depicted in Fig. 1A, the predominant phyla across the three groups are Bacteroidota, Pseudomonadota, and Bacillota. At the species level, Fig. 1B demonstrates that *Escherichia coli*, *Phocaeicola vulgatus*, *Bacteroides fragilis*, *uncultured bacterium*, *Phocaeicola dorei*, and *Enterobacter kobei* exhibit high abundance in all three groups. Notably, *Phocaeicola vulgatus* is more prevalent in the N_total group compared to the other two groups, whereas *Bacteroides fragilis* is more abundant in the C_total group than in the other groups, with the abundance of these species in the P_total group falling between the two. Subsequently, a β-diversity analysis was conducted to assess the differences in microbial structure among the three sample groups. The findings, presented in Fig. 1C, indicate that there are no statistically significant differences among the groups. However, the results of pairwise comparisons demonstrate that the microbial structures of the N_total and P_total groups are more similar, with an *R*^2^ value of 0.0078, which is smaller than the differences between the other groups (N_total vs. C_total, *R*^2^ = 0.0344; P_total vs. C_total, *R*^2^ = 0.0249). The taxonomic composition of the C_total group also deviates from the other two groups in terms of spatial structure. To further identify the significantly enriched microbes within each group, a Linear discriminant analysis effect size (LEfSe) analysis of the microbiomes was performed. The results indicated that the N_total group is significantly enriched with species such as *Alistipes putredinis*, *Cutibacterium acnes*, and *Bifidobacterium breve*. In the P_total group, the significantly enriched species include *Pseudomonas putida*, *Massilia* sp. NP310, and *Klebsiella pneumoniae*. By contrast, the C_total group showed significant enrichment only for *Malassezia restricta* when compared to the other two groups (Fig. 1D).

We analyzed the functional differences in the microbiome across three distinct groups. By examining the annotation results obtained from the Comprehensive Antibiotic Resistance Database (CARD), the Carbohydrate-Active Enzymes Database (CAZy), the Gene Ontology (GO), the evolutionary genealogy of genes: Non-supervised Orthologous Groups (eggNOG), the Kyoto Encyclopedia of Genes and Genomes (KEGG), the Virulence Factor Database (VFDB), and the Pfam database, we found that certain functions with higher abundance did not exhibit significant differences, despite variations among the groups (Fig. S1A through E). Specifically, the KEGG database annotations revealed that functions associated with the endocrine system and cell growth and death were more prevalent in normal mucosa and paracancerous tissue compared to cancerous tissue (Fig. S1A). Furthermore, the GO database annotations indicated a progressive increase in the relative abundance of cellular anatomical entities and catalytic activities across normal mucosa, paracancerous tissue, and cancerous tissue (Fig. S1B). Similarly, the VFDB database results demonstrated a gradual increase in immune modulation functions across these tissue types (Fig. S1C). Notably, the Pfam database annotations (Fig. 1E) revealed significant differences among the three groups. Among the highly abundant functional pathways, the relative abundance of Pilus subunit was significantly greater in the N_total and P_total groups compared to the C_total group. In addition, the relative abundance of globin-like, fumarate reductase respiratory complex transmembrane subunits, aspartate chemoreceptor, signal-transduction ligand-binding, and the 2H phosphoesterase superfamily was significantly higher in the N_total group relative to the C_total group. Moreover, we compared the KEGG data between the normal mucosal tissue N_total group and the cancer tissue C_total group (Fig. 1F). The results indicated that the differential functions were predominantly enriched in pathways such as the biosynthesis of antibiotics, the two-component system, carbon metabolism, and the biosynthesis of amino acids. These findings suggest that there are significant differences in the composition and functionality of microorganisms between cancerous tissue and both normal and paracancerous mucosa, with the disparities in microbiome functions being more pronounced in cancerous tissues compared to the other two tissue types.

### Microbiome of various tissue types in the right-sided colon and their functional differences

**Fig 2:**
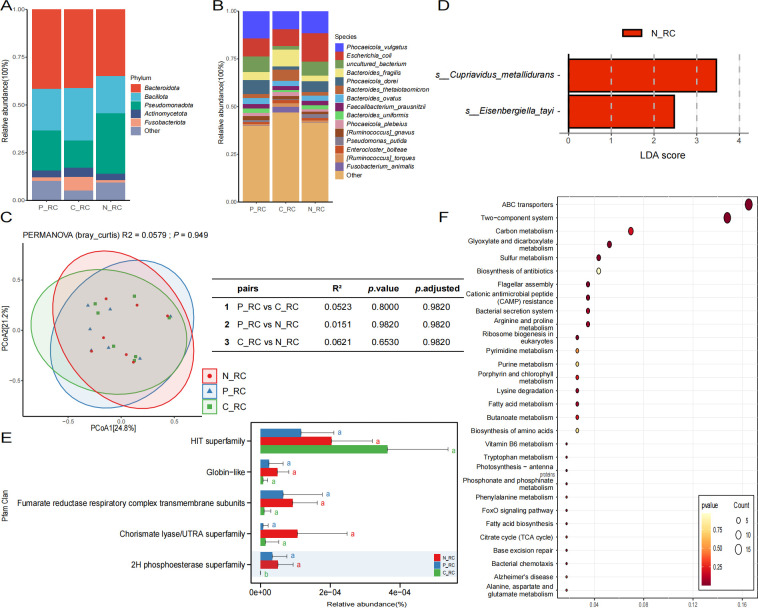
The microbiome analysis results from various types of tissues in the right-sided colon are presented as follows. Panels A and B display species composition bar charts at both the phylum and species levels. Panel C illustrates the results of the Bray-Curtis beta diversity analysis, where the shapes of different points correspond to distinct groups. Panel D presents the results of the LEfSe differential analysis, with the horizontal axis depicting the LDA score and the vertical axis indicating the type of microbial species. Panel E features a bar chart of Pfam functional differences, where the horizontal axis represents relative abundance, and the vertical axis indicates various functions; different letters between the two groups denote statistically significant differences, while the absence of differing letters indicates non-significance. As demonstrated in Panel F, the differential KEGG function bubble chart is presented. The horizontal axis denotes GeneRatio, with larger values indicating a greater total number of genes enriched in the pathway. The vertical axis represents different functions. The size of the circle corresponds to the number of counts enriched in the function, while the color of the circle signifies the *P*_value.

In light of the potential disparities in the pathogenesis of distinct intestinal locations, an exploratory study was conducted on tissue samples from diverse intestinal anatomical sites. Initially, the analysis encompassed various types of tissue samples from the right colon, which were categorized into three groups: normal mucosa (N_RC), paracancerous tissue (P_RC), and cancerous tissue (C_RC). The annotation results based on microbial taxonomy at the phylum level showed that the dominant phyla in the three groups were *Bacteroidota, Bacillota,* and *Pseudomonadota* (Fig. 2A). Among them, the proportion of *Bacillota* was higher in the C_RC group, and the proportion of *Pseudomonadota* was higher in the N_RC group. At the species level, the dominant species in the C_RC group were *Phocaeicola vulgatus, Escherichia coli, Bacteroides fragilis,* and *Bacteroides threoninaeae* (Fig. 2B). The dominant bacterial species in the N_RC and P_RC groups were *Phocaeicola vulgatus, Escherichia coli, uncultured bacterium,* and *Phocaeicola dore*. Further investigation into the β-diversity of the samples revealed no significant differences among the three groups (Fig. 2C). However, the findings of the paired test indicate that the N_RC group and the P_RC group demonstrate a relatively close proximity (*R*^2^ = 0.0151), while the discrepancy between the N_RC group and the C_RC group exhibits a marginally greater extent (*R*^2^ = 0.0621). To further explore the differential microbial markers between different tissue samples, Lefse analysis was performed on the differential species among the three groups. Utilizing an LDA threshold of >2, the analysis identified *Cupriavidus metallidurans* and *Eisenbergiella tayi* as being comparatively enriched in normal mucosal tissues (Fig. 2D).

Next, we explored the differential functions of the microbiomes among the three groups. The annotation results based on different databases showed that most of the differences between the groups did not reach a significant level, but there was still a certain trend of change in the microbiomes among the three groups. The annotation results based on the GO database showed that metabolic process, cellular process, cellular anatomical entity, binding, and catalytic activity functions had higher relative abundances than other GO categories. However, within these high-abundance pathways, we observed that the N_RC group exhibited a relative decrease in abundance compared to the other two groups (Fig. S2A). The annotation results, based on the KEGG database, demonstrated that the mean relative abundance of P_RC in the functional pathways associated with cell growth and death, signal transduction, folding, sorting, and degradation, infectious diseases: viral, endocrine, and metabolic diseases, cancers: specific types, cancers: overview, immune system, endocrine system, and development was higher than that observed in the other two groups (Fig. S2B). The annotation results based on the VFDB database demonstrated that the functions related to the effector delivery system were comparatively abundant in the N_RC group (Fig. S2C). Furthermore, the eggNOG database annotation results demonstrated that the functions of nucleotide transport and metabolism, cell cycle control, cell division, chromosome partitioning, and energy production and conversion were enriched in the N_RC group, while coenzyme transport and metabolism exhibited a higher abundance in the C_RC group (Fig. S2D). Annotation results from the CARD database revealed that penem, monobactam, and cephalosporin exhibited higher relative abundances in N_RC and P_RC compared to the other two groups (Fig. S2E). A similar trend was observed in the CAZy database, where the differences between the groups were minimal and the functions remained comparable (Fig. S2F). The annotation results based on the Pfam database showed that the HIT superfamily was relatively high in the C_RC group, the Chorismate lyase/UTRA superfamily was relatively high in the N_RC group, and the average relative abundance of Globin−like, Fumarate reductase respiratory complex transmembrane subunits, and the 2H phosphoesterase superfamily in the C_RC group was lower than that in the other two groups. Of particular note was the marked reduction in the 2H phosphoesterase superfamily in the C_RC group (Fig. 2E). Subsequent cluster analysis of the KEGG differential functions between the N_RC group and the C_RC group revealed that the differential functions between the two groups were predominantly enriched in the functional pathways related to ABC transporters and two-component systems (Fig. 2F).

### Microbiome of various tissue types in the left-sided colon and their functional differences

**Fig 3:**
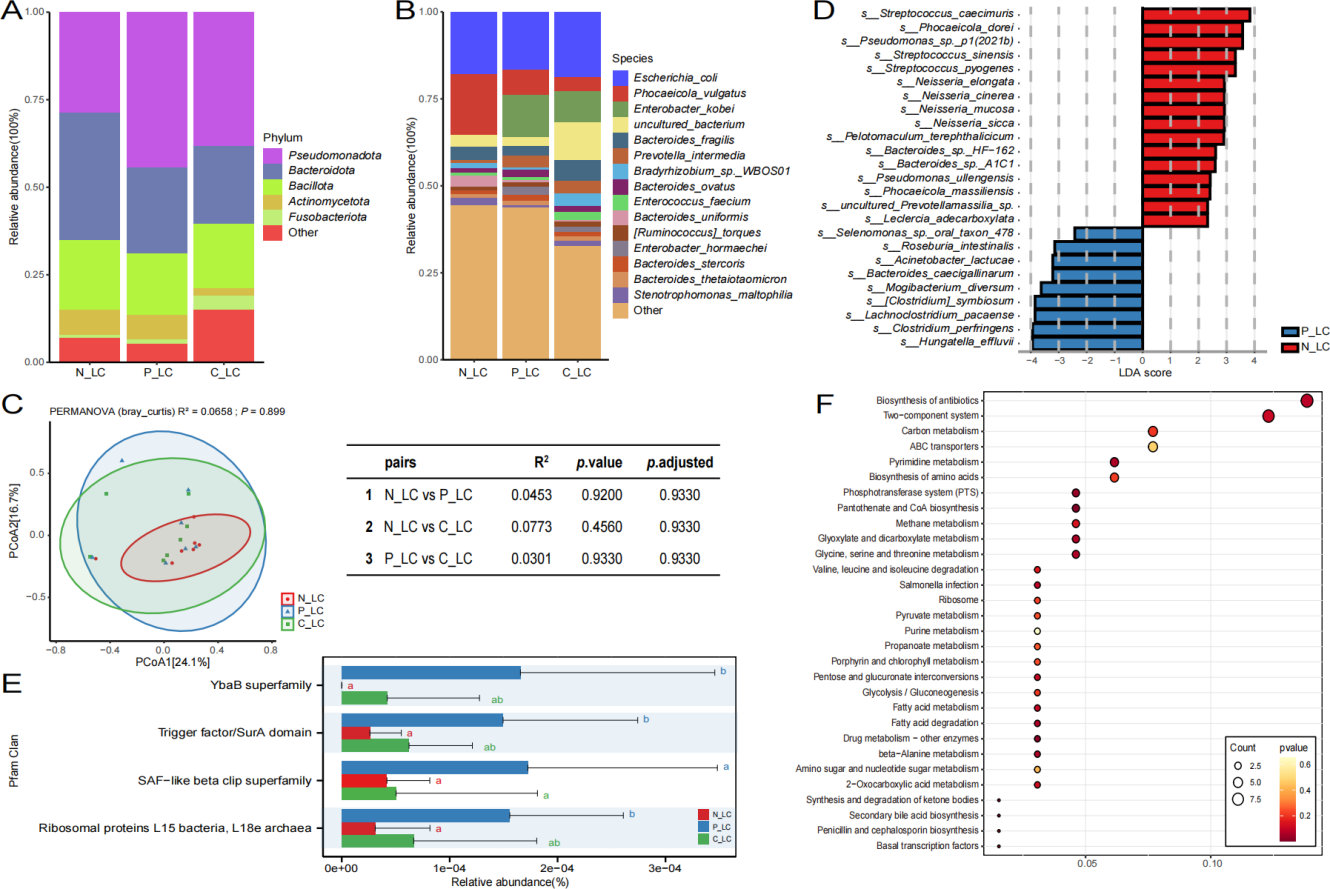
The microbiome analysis reveals the results from various tissue types in the left-sided colon. Panels A and B present bar graphs illustrating the composition of microbial communities at both the phylum and species levels. Panel C displays the Bray-Curtis β diversity analysis, with different shapes denoting distinct groups. Panel D showcases the results of LEfSe analysis, where the horizontal axis represents the LDA score and the vertical axis indicates different microbial species types. Panel E features a bar chart of Pfam functional differences, with the horizontal axis indicating relative abundance and the vertical axis representing various functions; significant differences between the two groups are denoted by different letters, while the absence of differing letters indicates no significant differences. As illustrated in panel F, the differential KEGG function bubble chart is displayed. The horizontal axis denotes GeneRatio, with larger values indicating a greater total number of genes enriched in the pathway. The vertical axis represents different functions. The size of the circle corresponds to the number of counts enriched in the function, while the color of the circle signifies the *P*_value.

In a similar vein, a thorough categorization and analysis of various types of tissues in the left colon were performed. These tissues were classified into three categories: normal mucosa (N_LC), paracancerous tissue (P_LC), and cancerous tissue (C_LC). Subsequently, we performed a differential analysis of their microbial composition and functional pathways. As illustrated in Fig. 3A, at the phylum level, the predominant phyla across the three groups were identified as *Pseudomonadota, Bacteroidota,* and *Bacillota*. At the species level (Fig. 3B), the dominant species in the N_LC group included *Escherichia coli* and *Phocaeicola vulgatus*, while the P_LC group was characterized by *Escherichia coli* and *Enterobacter kobei*. In the C_LC group, the dominant species were *Escherichia coli,* uncultured bacterium, and *Enterobacter kobei*. Notably, the proportion of *Phocaeicola vulgatus* was elevated in the N_LC group compared to the other two groups, whereas the proportion of *Enterobacter kobei* was reduced in comparison to the other groups. The results of the β diversity analysis, based on taxonomic composition, indicated no significant differences in microbial structure among the three groups (Fig. 3C). The results of the β diversity analysis, based on taxonomic composition, demonstrated that there was no significant difference in microbial structure among the three groups (Fig. 3C). Nevertheless, the results based on the pairwise test revealed that the difference between the C_LC group and the N_LC group was more pronounced (*R*^2^ = 0.0773) and, conversely, the difference between the P_LC group and the other two groups was less considerable (*R*^2^ = 0.0453, *R*^2^ = 0.0301). Subsequent lefse analysis of the data from the three groups was conducted to ascertain the differential microbial markers of each group. The final results demonstrated that, in comparison with the other two groups, no significantly enriched microorganisms were identified in the C_LC group. However, 16 significantly enriched microbial species were identified in the N_LC group, primarily *Streptococcus_caecimuris*, and 9 significantly enriched microorganisms were identified in the P_LC group, primarily *Hungatella_effluvii* (Fig. 3D).

The annotation outcomes derived from disparate databases exhibited congruence with those of the right colon subgroup analysis, with no substantial disparities observed in the majority of functions across the three groups. The annotation results based on the GO database demonstrated near-perfect concordance with the trend observed in the right colon. The GO category, encompassing metabolic process, cellular process, cellular anatomical entity, binding, and catalytic activity, was predominant across all groups. However, the N_LC group exhibited a relative deficiency in these functions when compared to the other two groups (Fig. S3A). The annotation results based on the KEGG database demonstrated that the signal transduction and replication and repair functions of the P_LC group were comparatively enriched (Fig. S3B). The annotation results based on the VFDB database demonstrated that nutritional/metabolic factors, immune modulation, and adherence were comparatively low in the N_LC group (Fig. S3C). In the annotation results of the eggNOG database, it was evident that the coenzyme transport and metabolism functions were comparatively enriched in the N_LC group (Fig. S3D). Furthermore, the annotation results of the CARD database revealed that the average relative abundance of penem, monobactam, and cephalosporin in the P_LC group was higher than that in the other two groups, which was consistent with the analysis results in the right colon (Fig. S3E). In the annotation results of the CAZy database, the trend indicated that glycosyltransferase was lower in the N_LC group (Fig. S3F). Furthermore, the annotation results of the Pfam database revealed that the YbaB superfamily, trigger factor/SurA domain, and ribosomal proteins L15 bacteria and L18e archaea exhibited lower levels in the N_LC group, reaching a significantly different level from that of the P_LC group (Fig. 3E). In addition, a cluster analysis was performed on the KEGG differential functions between the N_LC group and the C_LC group. The results showed that the differential functions between the two groups were mainly concentrated in the biosynthesis of antibiotics and the two-component system (Fig. 3F).

### Differences in the microbiomes of left and right colon tissues

**Fig 4:**
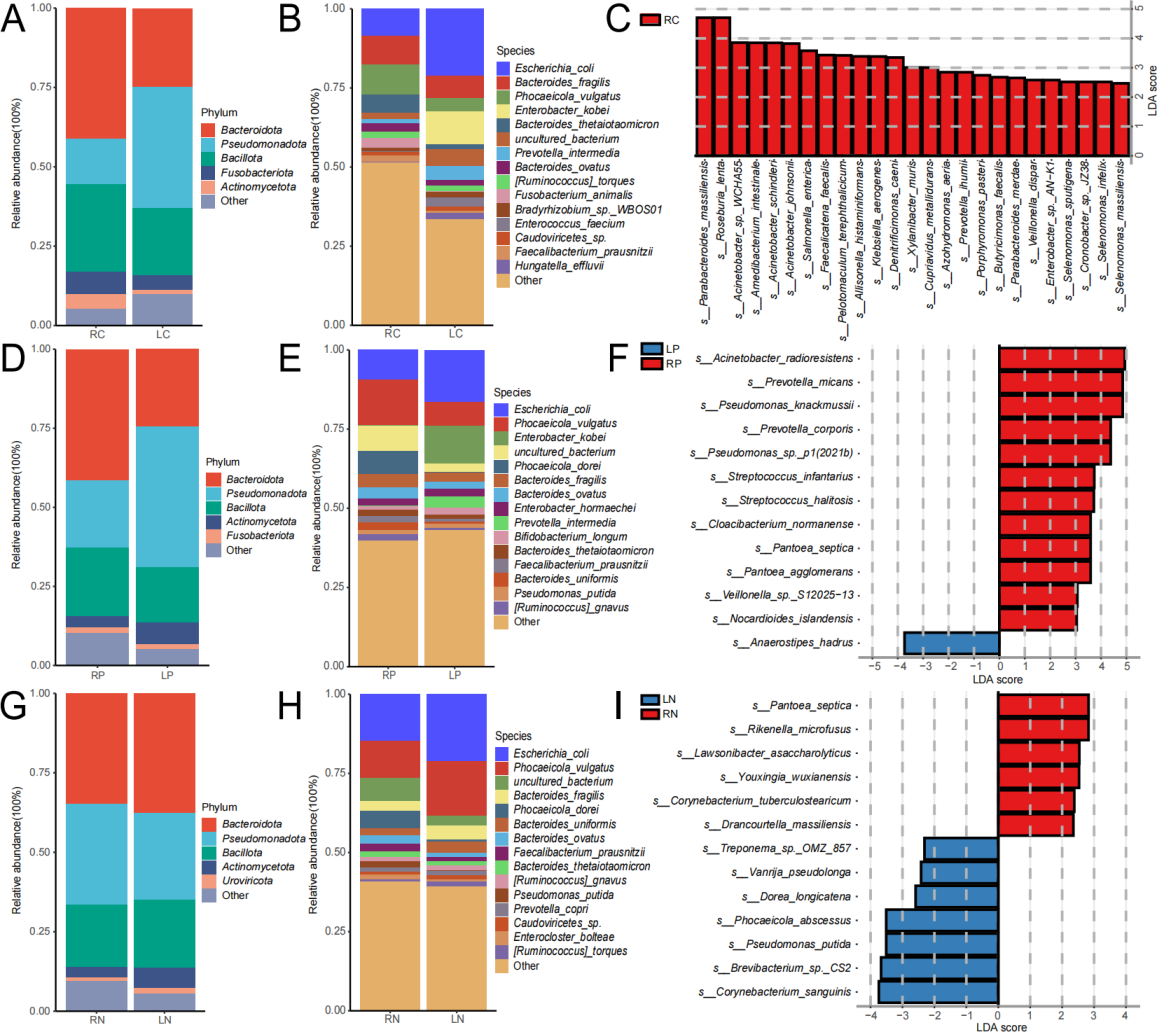
The analysis results of the microbiome from various tissue types in the left and right colon are presented. Panels A, B, D, E, G, and H display bar charts illustrating the taxonomic composition at both the phylum and species levels, while panels C, F, and I show the results of LEfSe differential analysis, with the horizontal axis indicating LDA scores and the vertical axis representing the different types of microbial species.

Next, we systematically investigated the microbiome data from the left and right colon tissues to elucidate how these differences may influence colorectal cancer, to offer more precise diagnostic and therapeutic guidance for clinical practice. Our comparison of α diversity between the two groups revealed no significant differences in microbial diversity between the left colon (LC) and right colon (RC) cancer tissues (Fig. S4A). In addition, β diversity analyses indicated no significant differences in the composition of the microbiomes between the two groups (Fig. S4B). Taxonomic composition analysis showed that the cancer tissue in the left colon is predominantly comprised of *Pseudomonas* and *Bacteroidota*, whereas the right colon cancer tissue primarily consists of *Bacteroidota* and *Bacillota* (Fig. 4A). At the species level, the left colon cancer tissue is largely characterized by the presence of *Escherichia coli, Bacteroides fragilis*, and *Enterobacter kobei*, while the right colon cancer tissue is mainly composed of *Phocaeicola vulgatus, B. fragilis*, and *E. coli* (Fig. 4B). Notably, *E. kobei* and *E. coli* are relatively enriched in the cancer tissue of the left colon. Further analysis using LefSe identified 25 species, including *Parabacteroides massiliensis* and *Roseburia lenta*, that are significantly enriched in the right colon cancer tissue, while no significant differential microbial biomarkers were identified in the left colon cancer tissue (Fig. 4C). Functionally, the Pfam functional annotation results demonstrated a significant increase in the relative abundance of probable substrate-binding ATP-grasp domains, PHM/PNGase F superfamily components, and heme NO and oxygen-binding-like domains in the right colon cancer tissue (Fig. S4C). Moreover, functional annotation based on the CARD database revealed that penam-related functions are significantly enriched in left colon cancer tissues (Fig. S4D). However, functional analyses conducted using other databases failed to uncover any significant differences in functional pathways between the two groups (Fig. S4E through I).

In comparing paracancerous tissues from the left and right colon, no significant differences in α and β diversity were observed between the left peritumoral tissue (LP) and the right peritumoral tissue (RP) (Fig. S5A and B). Regarding species composition, the right peritumoral tissue was predominantly dominated by the phylum *Bacteroidota*, whereas the left peritumoral tissue was mainly characterized by *Pseudomonadota* (Fig. 4D). At the species level, LP was primarily comprised of *E. coli*, *P. vulgatus*, and *E. kobei*, while RP was mainly composed of *E. coli*, *P. vulgatus*, and *uncultured bacteria*. Consistent with the findings in left and right colon cancer tissue samples, *Escherichia coli* exhibited relative abundance in the left colon peritumoral tissue (Fig. 4E). LefSe analysis revealed that *Anaerostipes hadrus* was significantly enriched in the LP group, whereas 12 species, including *Acinetobacter radioresistens*, *Prevotella micans*, and *Pseudomonas knackmussii*, were significantly enriched in the RP group (Fig. 4F). Functionally, annotation results based on the Pfam database indicated significant enrichment of pathways related to the trigger factor/SurA domain, DNA polymerase B-like, and Aha1/BPI domain-like superfamily in the LC group (Fig. S5C). However, comparative analyses of annotation results from other databases revealed no significant differences (Fig. S5D through I).

Finally, comparative analyses of left normal tissue (LN) and right normal tissue (RN) indicated no significant differences between the two groups regarding α and β diversity (Fig. S6A and B). Species composition proportions also showed no notable differences between the two groups at the phylum level. Although no significant variations were observed among dominant species at the species level, *E. coli* and *P. vulgatus* exhibited slightly higher proportions in the LN group (Fig. 4G and H). LefSe analysis indicated that six species, including Pantoea septica and *Rikenella microfusus*, were significantly enriched in the RN group, while seven species, including *Corynebacterium sanguinis* and *Brevibacterium* sp. CS2, were significantly enriched in the LN group (Fig. 4I). Comparative functional results demonstrated that annotations based on the Pfam database indicated significant enrichment of the type III secretion system domain superfamily and EF-hand-like superfamily in the RN group (Fig. S6C), with no significant differences found in analyses of annotations from other databases (Fig. S6D through I).

### Microbiome of various tissue types in the rectum and their functional differences

**Fig 5:**
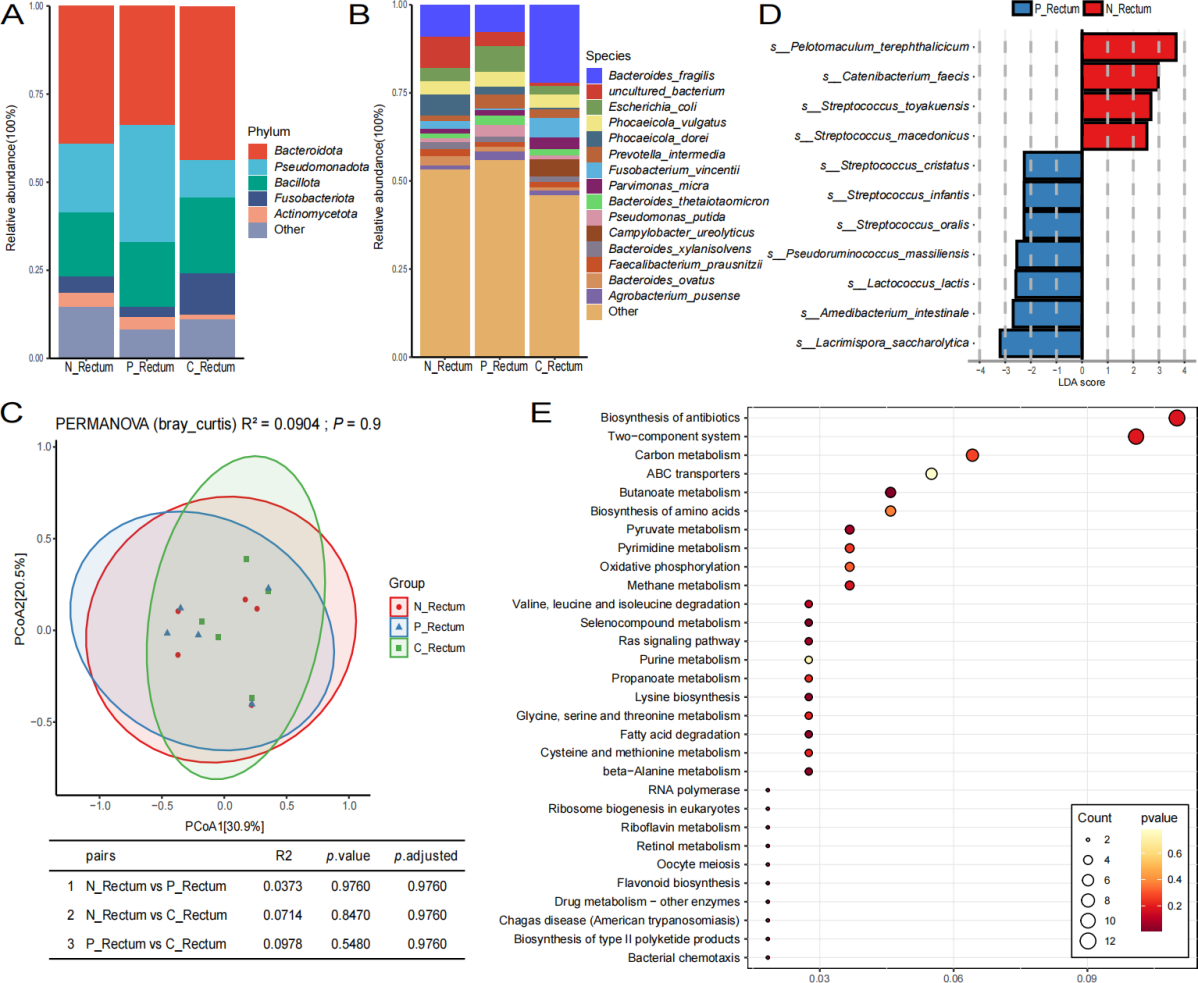
The microbiome analysis results for various types of rectal tissues are presented as follows: Panels A and B display bar charts illustrating species composition at the phylum and species levels. Panel C shows the results of Bray-Curtis β-diversity analysis, with the shapes of different points representing distinct groups. Panel D presents the Lefse difference analysis, where the horizontal axis indicates the LDA score and the vertical axis denotes the type of microbial species. As illustrated in panel E, the differential KEGG function bubble chart is presented. The horizontal axis denotes GeneRatio, with larger values indicating a greater total number of genes enriched in the pathway. The vertical axis represents different functions. The size of the circle corresponds to the number of counts enriched in the function, while the color of the circle signifies the *P*_value.

Similarly, subgroup analysis was performed for different types of rectal tissue according to normal mucosa (N_Rectum), paracancerous tissue (P_Rectum), and cancerous tissue (C_Rectum). At the phylum level, consistent with the results of the previous subgroup analysis, the dominant phyla in the three groups were *Bacteroidota, Bacillota,* and *Pseudomonadota* (Fig. 5A). At the species level, the dominant species in the C_Rectum group were *Phocaeicola vulgatus, Fusobacterium vincentii,* and *Bacteroides fragilis,* while the dominant species in the N_Rectum group were *Bacteroides fragilis, uncultured bacterium,* and *Phocaeicola dorei*. The most prevalent species in the P Rectum group were *Phocaeicola vulgatus, Escherichia coli, Prevotella intermedia*, and *Bacteroides fragilis* (Fig. 5B). The results of the beta diversity analysis of species taxonomic annotation revealed that there was no significant difference in beta diversity among the three groups. However, the N_Rectum group and the P_Rectum group were found to be closely related, while the C_Rectum group exhibited a large difference (Fig. 5C). Subsequent Lefse analysis of differential species among the three groups revealed that *Pelotomaculum terephthalicicum, Catenibacterium faecis, Streptococcus toyakuensis,* and *Streptococcus macedonicus* were enriched in normal mucosal tissues. Conversely, *Lacrimispora saccharolytica, Amedibacterium intestinale,* and *Lactococcus lactis* were found to be enriched in paracancerous tissues (Fig. 5D).

The results of the analysis based on functional pathways were similar to those before, and the microbial functions among the three groups did not reach a significant level of difference. The annotation results based on the GO database also showed that metabolic process, cellular process, cellular anatomical entity, binding, and catalytic activity dominated the functions, and they were all relatively abundant in the C_Rectum group (Fig. S7A). In the annotation results based on the VFDB database, immune modulation exhibited a higher trend in the C_Rectum group (Fig. S7B). In the annotation results based on the CARD database, a similar trend was observed as in the previous subgroups, and the relative abundance of penem, monobactam, and cephalosporin was higher in N_Rectum and P_Rectum (Fig. S7C). The annotation results based on the Pfam database demonstrated that the average relative abundance of the transporter-like superfamily, four TM region, exhibited a higher tendency in the N_Rectum and P_Rectum groups (Fig. S7D). In the annotation results of the KEGG, CAZy, and eggNOG databases, the functional abundances among the three groups were found to be relatively similar (Fig. S7E through G). A cluster analysis was performed on the KEGG differential functions between the N_Rectum group and the C_Rectum group, revealing that the differential functions between the two groups were mainly concentrated in the biosynthesis of antibiotics and the two-component system (Fig. 5E).

### Random forest prediction of significant microorganisms associated with CRC

**Fig 6:**
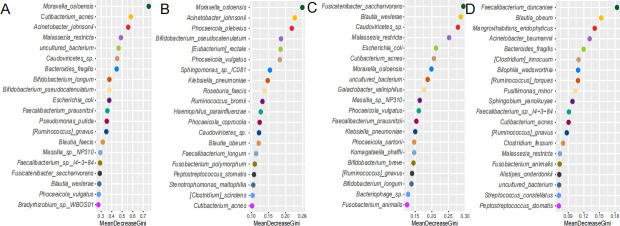
The results of the random forest predictions for key microorganisms associated with colorectal cancer are presented in this study. Panel A displays the random forest prediction results for the overall colorectal grouping, while panel B focuses on the right-sided colon grouping, panel C focuses on the left-sided colon grouping, and panel D focuses on the rectal grouping. The horizontal axis represents the mean decrease Gini value, where a higher value indicates greater importance, and the vertical axis includes various species analyzed in the predictions.

The present study utilized a random forest prediction model to analyze data to obtain significant microorganisms associated with CRC. The random forest analysis of the data from the three groups of N_total, P_total, and C_total (Fig. 6A) revealed that the species *Moraxella osloensis* was the most significant in terms of predicting the classification of these three groups. This finding suggests that it can be utilized as a potential indicator for the clinical diagnosis and treatment of CRC. The same analysis was also performed on subgroups of different anatomical parts of the intestine, and it was found that *Moraxella osloensis* in the right-sided colon was also the most significant microbial distinguishing factor between different tissue samples (Fig. 6B). In the analysis based on the left-sided colon, *Fusicatenibacter saccharivorans* was found to be of certain importance in predicting the health of the left-sided colon (Fig. 6C). Finally, in the subgroup analysis based on rectum location, *Faecalibacterium duncaniae* was shown to be an important species for predicting rectal health status (Fig. 6D). These analyses provide reference indicators for clinical prediction of the health status of different parts of the colorectum.

## DISCUSSION

It is widely accepted that many intestinal bacteria play a pivotal role in various inflammatory and immune processes that influence tumor etiology. This is attributable to their metabolic properties and their capacity to modify the equilibrium of the microbiome, which is instrumental in maintaining bodily health. In the present study, 57 colorectal tissue samples were categorized into normal mucosa, paracancerous tissue, and cancerous tissue. Metagenomic sequencing was employed to elucidate alterations in their microbiome. Following the implementation of quality control procedures and the elimination of host contamination, an in-depth analysis of the data was conducted, ensuring the fulfillment of the criteria necessary for subsequent analysis. Our analysis revealed that the three types of tissues are abundant in the same microbial phyla, *Bacteroidota, Pseudomonadota,* and *Bacillota*. This finding suggests that colorectal cancer does not result in significant alterations in the microbial composition at the phylum level. Following in-depth analysis at the species level, it was determined that although the dominant species among the three tissues are similar, their abundance ratios vary significantly, and the abundance of the microbial community in the paracancerous tissue is between that of normal mucosa and cancerous tissue, a finding that is also reflected in the analysis results of β diversity. Further differential analysis of these altered species revealed that *Alistipes putredinis, Cutibacterium acnes,* and *Bifidobacterium breve* were significantly enriched in normal mucosa. *Alistipes* are anaerobic bacteria found mostly in the healthy human gastrointestinal tract microbiota (14). There is evidence to suggest that *Alistipes* may have a protective effect against certain diseases, including liver fibrosis, colitis, cancer immunotherapy, and cardiovascular disease (14). There are also reports that *Alistipes putredinis* can provide energy for intestinal epithelial cells by producing short-chain fatty acids and may have anti-inflammatory properties (15, 16). It should be noted, however, that *Alistipes* may be associated with a risk of colorectal cancer, as evidenced in a study by Moschen et al. (17). *Cutibacterium acnes* is a significant species within the human microbiome. It is a gram-positive, non-spore-forming, anaerobic/aerotolerant rod-shaped bacterium that is primarily located in sebaceous glands and hair follicles but can also be found in the oral cavity and gastrointestinal tract, upper respiratory tract, and urinary tract (18, 19). Previous reports on this bacterium demonstrated its propensity to aggregate on skin, a process that has been linked to the mitigation of oxidative stress and the preservation of skin health (19). However, the literature on *Cutibacterium acnes* and colorectal cancer remains limited. The findings of this study suggest a potential association between the bacterium and colorectal health. *Bifidobacterium* is classified as a gram-positive, anaerobic, non-motile, non-spore-forming, sugar-degrading bacterium (20). Its extracellular structures, secretions, and bioactive metabolites have been shown to possess the capacity to impede the proliferation of potentially harmful bacteria, thereby promoting the survival of intestinal epithelial cells. Moreover, it has been demonstrated to regulate the host’s immune system. Its potential in cancer immunotherapy is promising, as evidenced by recent studies (21). This study found that *Bifidobacterium breve*, enriched in normal mucosa, may have a certain effect on the treatment or resistance of cancer, and further verification experiments can be carried out. The bacterial species that are enriched in paracancerous tissue include *Pseudomonas putida, Massilia sp*. NP310, and *Klebsiella pneumoniae. Pseudomonas putida* has been reported to be widely present in soil, water sources, and the environment. It is a beneficial soil microorganism that can decompose a variety of organic substances, but it is not a major component of normal human microorganisms (22). The bacterium has been reported as a conditional pathogen, which can cause adverse effects when the human body’s resistance is poor (23). Infections caused by *Pseudomonas putida* mainly occur in immunocompromised individuals, including those with neutropenia, newborns, and cancer patients (24–26). *Massilia* is a ubiquitous organism, found in soil, the rhizosphere of plants, the atmosphere, water, and other environments. It has been demonstrated to promote plant growth (27, 28). There is a paucity of research on its role in human health, although it has been reported to be enriched in the intestines of patients with pancreatic head cancer (29). *Klebsiella pneumoniae* is a bacterium that is commonly present in the human population. However, it is susceptible to infection in individuals with compromised or weakened immune systems and has been linked to a range of diseases (30, 31). By contrast, *Malassezia restricta* is enriched in cancerous tissues and has been linked to Crohn’s disease, with the potential to exacerbate intestinal inflammation (32). Furthermore, an investigation into the enriched presence of *Malassezia* in pancreatic ductal adenocarcinoma (PDA) tumor tissues revealed that it promoted tumor growth by activating the complement pathway, involving mannose-binding lectins (33). This study also found the enrichment of *Malassezia restricta* in cancer tissues, suggesting that its dysregulation may be related to the occurrence and development of certain cancers. Furthermore, the composition of microorganisms has been shown to change, with altered functions being observed, and the differential functions of microorganisms between normal mucosa and cancerous tissues are enriched in pathways such as biosynthesis of antibiotics, two-component system, carbon metabolism, and biosynthesis of amino acids. This suggests that bacteria may affect the health of the host through differences in these functions.

Furthermore, while the right-sided colon, left-sided colon, and rectum are all components of the large intestine, it is evident that distinct anatomical locations within the intestinal tract can significantly influence the composition of the intestinal microbiota (34, 35). Moreover, there are notable differences in clinical manifestations, molecular characteristics, and treatment approaches between right-sided and left-sided colon cancer (34, 35). Consequently, this study conducted a comprehensive analysis of tissue microorganisms across various anatomical locations within the large intestine samples. The results revealed variability in alterations of the microbiome between cancerous tissue and normal mucosa as well as paracancerous tissue in the right-sided colon, left-sided colon, and rectum. Specifically, *Cupriavidus metallidurans* and *Eisenbergiella tayi* were predominantly present in the normal mucosa of the right-sided colon, *Streptococcus caecimuris* was primarily found in the left-sided colon, and *Pelotomaculum terephthalicicum, Catenibacterium faecis, Streptococcus toyakuensis,* and *Streptococcus macedonicus* were predominantly identified in the rectum. In paracancerous tissues, no significantly enriched species were observed in the right-sided colon, whereas *Hungatella effluvii* was enriched in the left-sided colon. Conversely, *Lacrimispora saccharolytica, Amedibacterium intestinale,* and *Lactococcus lactis* showed enrichment in the rectal tissue. In cancerous tissues, no significantly enriched species were identified across any of the subgroups. Although the composition of microbial communities varies between tissues in different anatomical regions of the intestine, the functional roles of these communities appear to be relatively similar, suggesting that cancerous and non-cancerous tissues in distinct regions may harbor different bacterial species that influence host health through analogous mechanisms. Nonetheless, given the relatively limited sample sizes in the subgroups, a larger data set may be necessary to validate these findings and enhance the reliability of the results.

To identify potential therapeutic targets for clinical medicine, this study established a model based on random forest analysis to predict microbial targets most relevant to health and cancer. The results indicated that, following the grouping of all samples into normal mucosa, paracancerous tissue, and cancerous tissue, the most important microbial species distinguishing these three groups was *Moraxella osloensis*. Although *Moraxella osloensis* is recognized as a conditional pathogen that typically does not cause disease, its surface components have been reported to induce mucosal inflammation in immunocompromised adults (36, 37). Nevertheless, its precise role in colorectal cancer remains to be fully elucidated. Subsequent random forest modeling of the data from each subgroup revealed that *Moraxella osloensis* is significant for determining the health status of the right-sided colon, while *Fusicatenibacter saccharivorans* is significant for assessing the health status of the left-sided colon, and *Faecalibacterium duncaniae* is crucial for the health status of the rectal mucosa. *Fusicatenibacter* has been identified as a beneficial genus capable of producing short-chain fatty acids (38), yet research on its relationship with colorectal cancer remains limited. In a study on intestinal microecological transplantation for the treatment of chronic radiation enteritis, *Fusicatenibacter saccharivorans* was found to be more abundant in healthy donors (39). Previous studies have demonstrated that *Faecalibacterium* correlates positively with the efficacy of cancer immunotherapy and negatively with cancer incidence (39, 40). Furthermore, microorganisms from this genus have been shown to produce short-chain fatty acids and are also linked to the occurrence and development of inflammatory bowel disease (41, 42). Numerous studies have demonstrated that gut microbiota and their metabolites significantly influence the occurrence and progression of colorectal-related cancers (43–45), prompting extensive research into the use of fecal microbiota detection for colorectal cancer screening (46–48). It is important to acknowledge that microorganisms primarily exert their effects by stimulating the mucosal tissue (49). This study offers a comprehensive and systematic analysis of the microbiomes present in healthy tissue, cancerous tissue, and paracancerous tissue of patients, thereby contributing to the advancement of precision early screening for colorectal cancer based on microbiome analysis.

In summary, this study utilized metagenomic sequencing to identify tissue-enriched bacterial flora across various anatomical structures of the intestine by analyzing the bacterial communities present in the normal mucosa, paracancerous tissue, and cancerous tissue of CRC patients. It described the microbiome characteristics of these three tissue types, provided insights into microbiota with potential protective or pathogenic effects, and suggested new potential targets for the clinical diagnosis and treatment of colorectal cancer.

## Data Availability

The data supporting the results of this study are available from the corresponding author on reasonable request. Raw data have been deposited in the National Center for Biotechnology Information (NCBI) with study no. PRJNA1245618.

## SUPPLEMENTAL MATERIAL

Text S1 msystems.00198-25-s0001.docx Detailed description of the definition of RC and LC and the methods for sample collection.

Figure S1 msystems.00198-25-s0002.tif The functional differences in the microbiome across the normal mucosa, paracancerous tissue, and cancerous tissue.

Figure S2 msystems.00198-25-s0003.tif The differential functions of the microbiomes among various tissue types in the right-sided colon.

Figure S3 msystems.00198-25-s0004.tif The differential functions of the microbiomes among various tissue types in the left-sided colon.

Figure S4 msystems.00198-25-s0005.tif Differences in microbiomes of left and right colon cancerous tissues.

Figure S5 msystems.00198-25-s0006.tif Differences in microbiomes of left and right colon paracancerous tissues.

Figure S6 msystems.00198-25-s0007.tif Differences in microbiomes of left and right colon normal tissues.

Figure S7 msystems.00198-25-s0008.tif The results of the analysis based on functional pathways in the rectum.

Legends msystems.00198-25-s0009.docx Supplemental figure legends.

Supplemental Tables msystems.00198-25-s0010.xlsx Tables S1 and S2.

ASM does not own the copyrights to Supplemental Material that may be linked to, or accessed through, an article. The authors have granted ASM a non-exclusive, world-wide license to publish the Supplemental Material files. Please contact the corresponding author directly for reuse.
