# Supplementary material for: The characteristics of tissue microbiota in different anatomical locations and different tissue types of the colorectum in patients with colorectal cancer
Source: mSystems. 2025 May 27;10(6):e00198-25. doi: 10.1128/msystems.00198-25 (PMC12172456; doi:10.1128/msystems.00198-25)
Supplement: Text S1 — Detailed description of the definition of RC and LC and the methods for sample collection. [file msystems.00198-25-s0001.docx]

## Instructions for Collecting Tissue Samples from the Colon and Rectum of Patients with Colorectal Cancer

This study selected 19 CRC patients who underwent surgical treatment in the Gastrointestinal Surgery Department of the Central Hospital of Wuhan from February 2023 to July 2023. All patients had not received radiotherapy or chemotherapy before the surgery. The surgical resection samples were cut open 10 minutes after being removed from the body with sterile scissors on the opposite side of the mesentery, and 0.9% sodium chloride solution was used to rinse them clean. Sterile scissors or blades were used to successively collect normal mucosa (N), mucosa adjacent to the cancer (P), and cancer tissue (C), and they were immediately placed in collection tubes containing DNA extraction solution, frozen in liquid nitrogen tanks, and transferred to -80°C refrigerators for storage.
According to the tissue types, they were divided into three groups: normal mucosa group (N_total) 19 cases, mucosa adjacent to the cancer group (P_total) 19 cases, and cancer tissue group (C_total) 19 cases; according to the tumor location and surgical resection range, they were divided into three groups: right colon (RC) 7 cases, left colon (LC) 7 cases, and rectum (Rectum) 5 cases.
Normal mucosa: 10 cm or more proximal to the cancer focus in the surgical specimen was lifted with sterile forceps, and 2 pieces of mucosa were cut off with sterile scissors.
Paracancerous tissue: Sterilized and washed the forceps and scissors, and 2 pieces of mucosa were cut off from the proximal end of the surgical specimen within 1-2 cm from the edge of the cancer lesion with sterile forceps.
Cancerous tissue: Sterilized and washed the forceps, and along the longitudinal axis of the intestinal tube parallel to the most deeply infiltrated area of the cancer focus as judged by the naked eye, 2 thin slices of cancer tissue were taken from the parallel side of the one side of the incision margin with an sterile knife.
Right-sided colon (RC), including the caecum, ascending colon, proximal 1/2 of the transverse colon; left-sided colon (LC), including the distal 1/2 of the transverse colon, descending colon, sigmoid colon; the rest was rectum (Rectum).
N_RC, P_RC, C_RC were the normal mucosa group, mucosa adjacent to the cancer group, and cancer tissue group of the right-sided colon group respectively. N_LC, P_LC, C_LC were the normal mucosa group, mucosa adjacent to the cancer group, and cancer tissue group of the left-sided colon group respectively. N_Rectum, P_Rectum, C_Rectum were the normal mucosa group, mucosa adjacent to the cancer group, and cancer tissue group of the rectum group respectively.


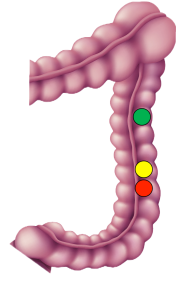

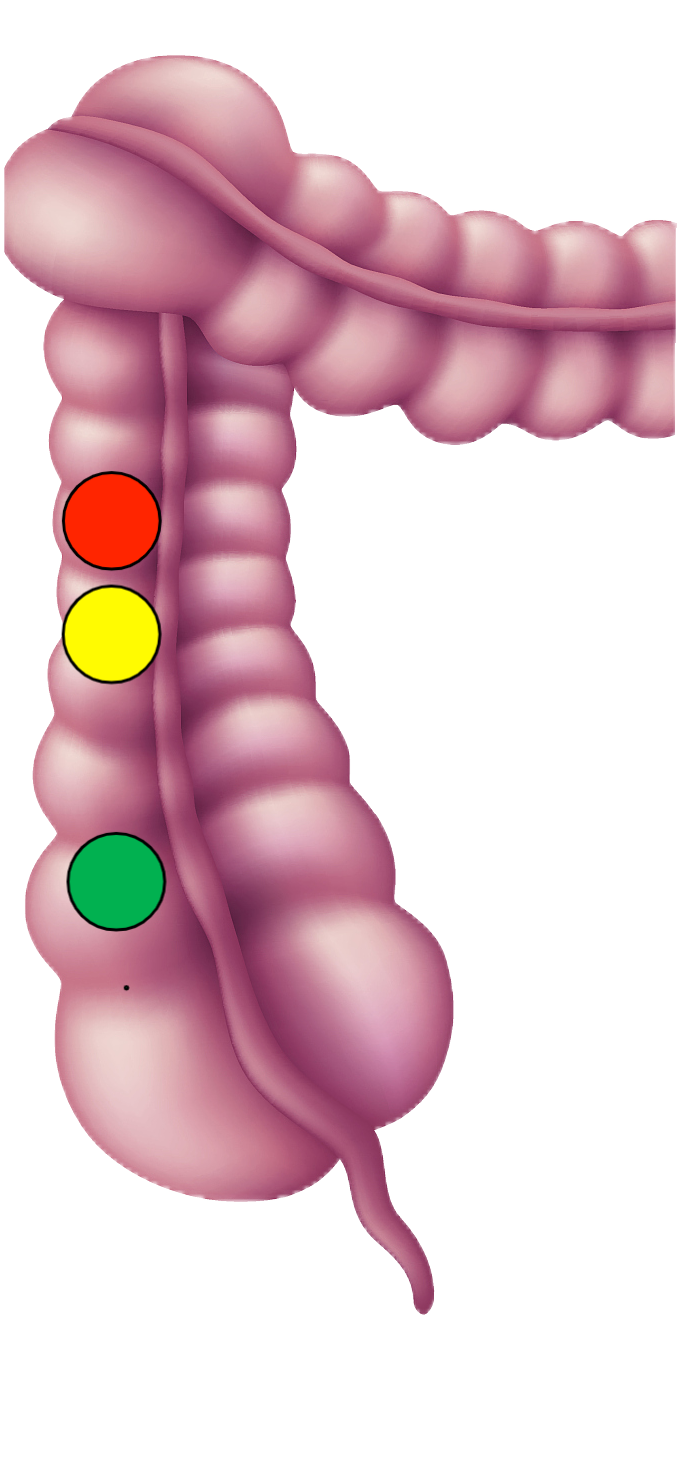

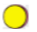

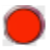

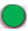

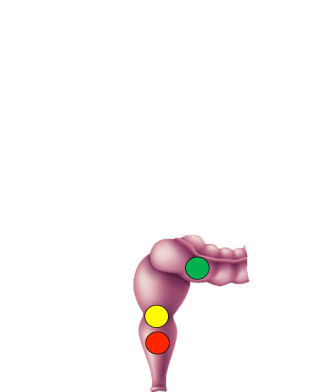

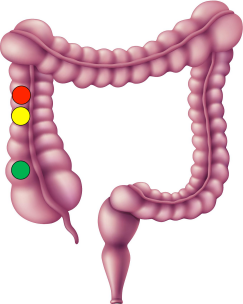


Rectum

Right-sided colon

Colorectum

Paracancerous tissue

Cancerous tissue

Normal mucosa

Left-sided colon
