# Supplementary figures and images for: The characteristics of tissue microbiota in different anatomical locations and different tissue types of the colorectum in patients with colorectal cancer
Source: mSystems. 2025 May 27;10(6):e00198-25. doi: 10.1128/msystems.00198-25 (PMC12172456; doi:10.1128/msystems.00198-25)

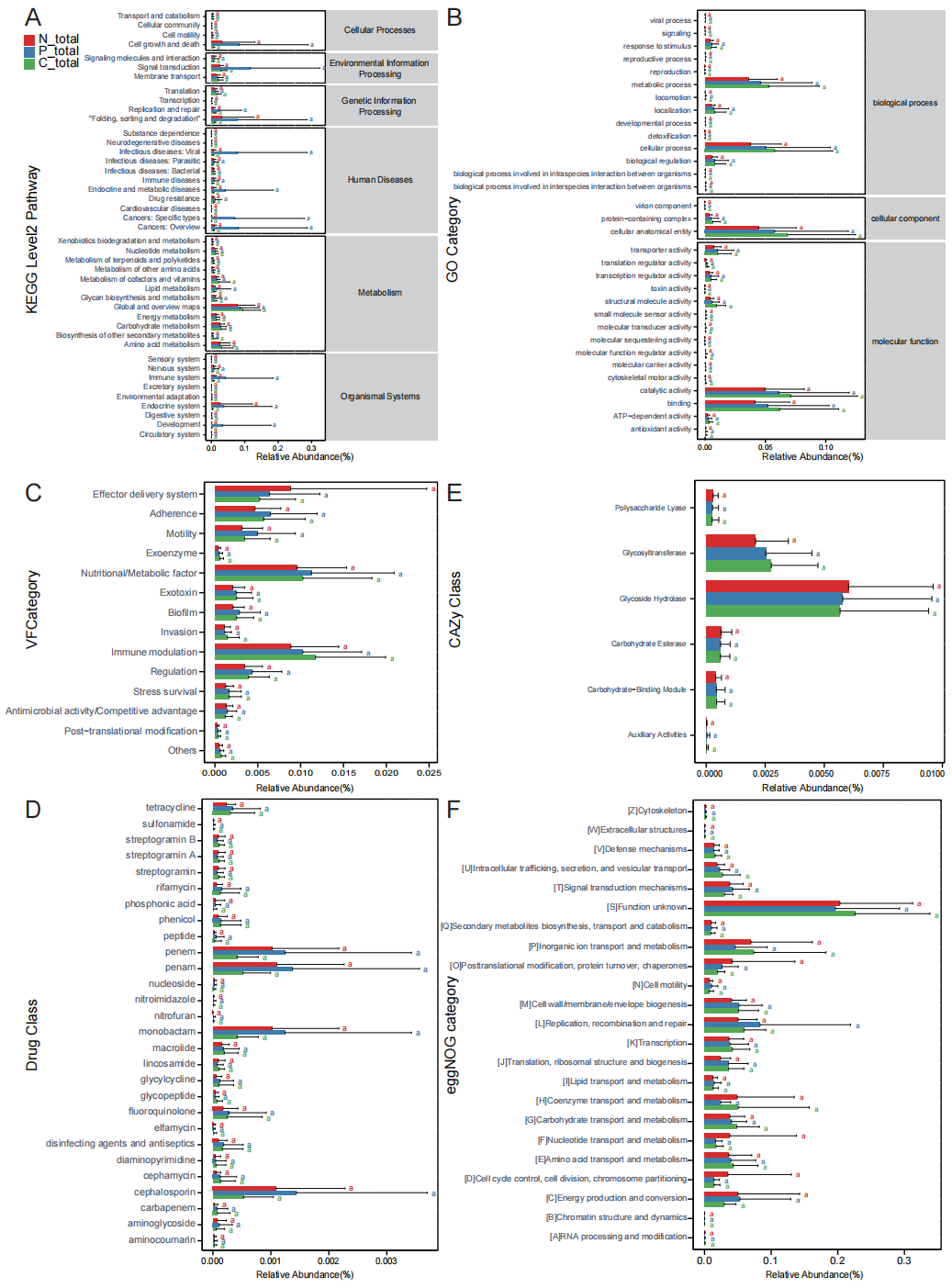

Supplement: Figure S1 — The functional differences in the microbiome across the normal mucosa, paracancerous tissue, and cancerous tissue. [file msystems.00198-25-s0002.tif]

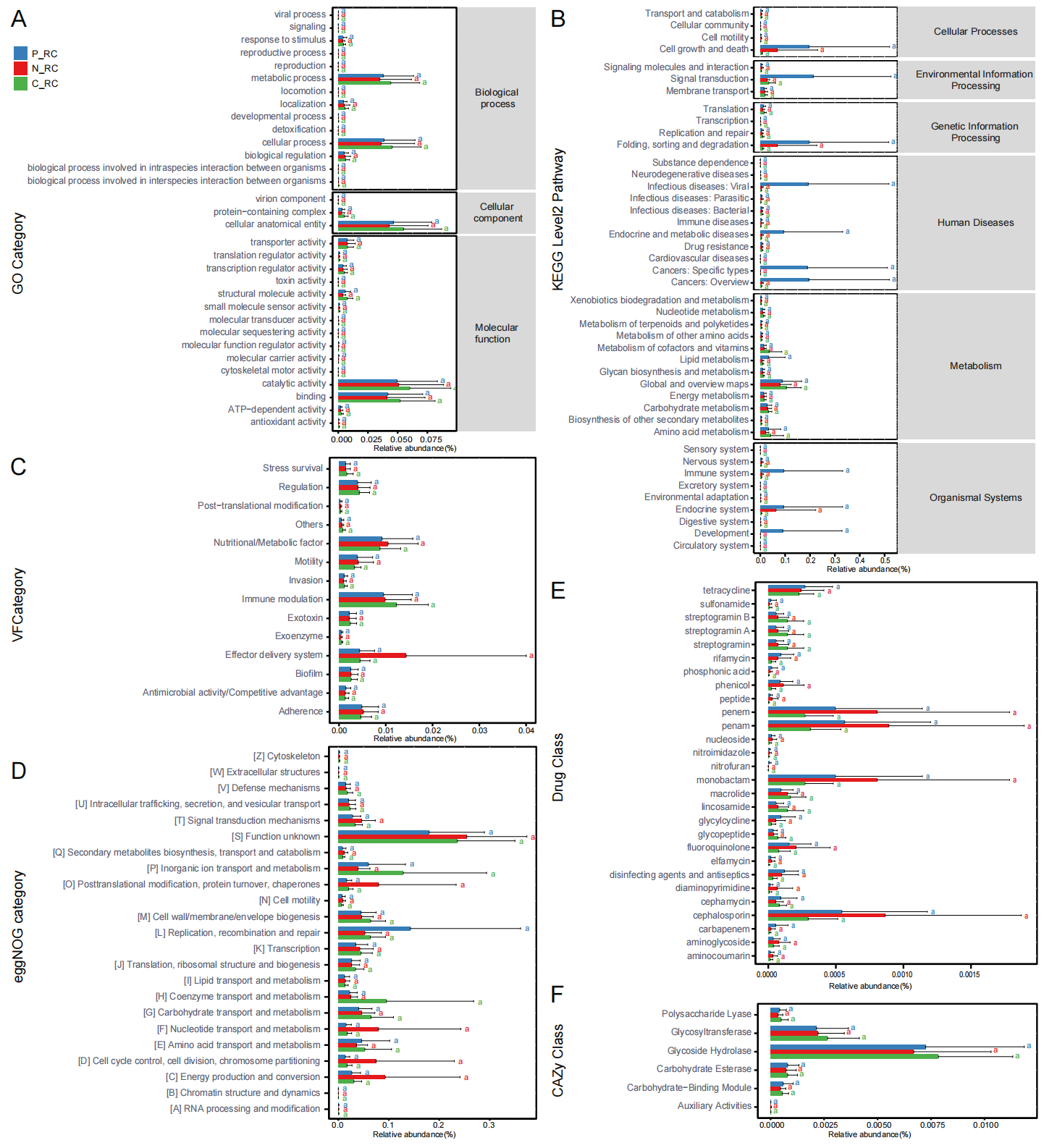

Supplement: Figure S2 — The differential functions of the microbiomes among various tissue types in the right-sided colon. [file msystems.00198-25-s0003.tif]

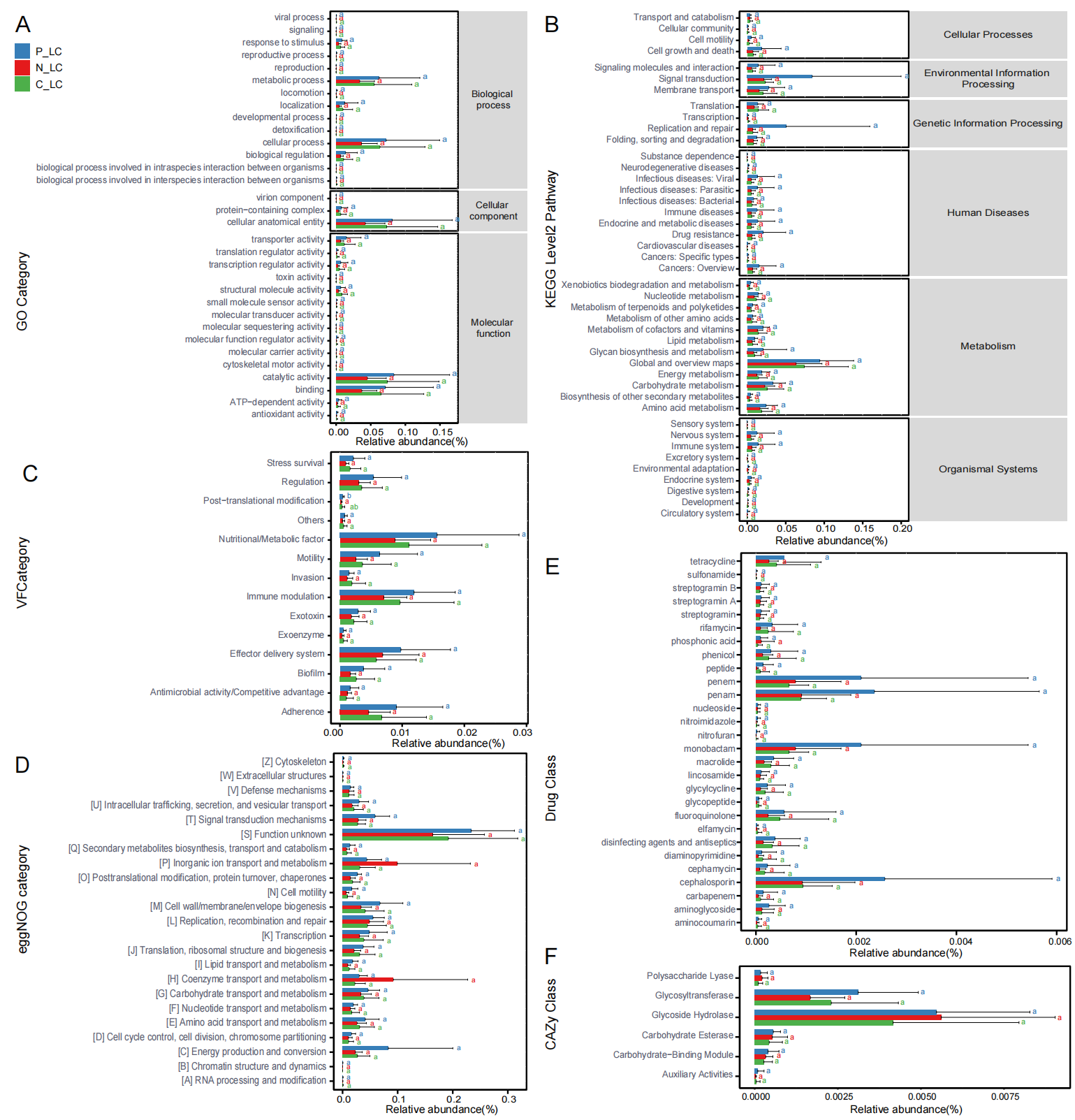

Supplement: Figure S3 — The differential functions of the microbiomes among various tissue types in the left-sided colon. [file msystems.00198-25-s0004.tif]

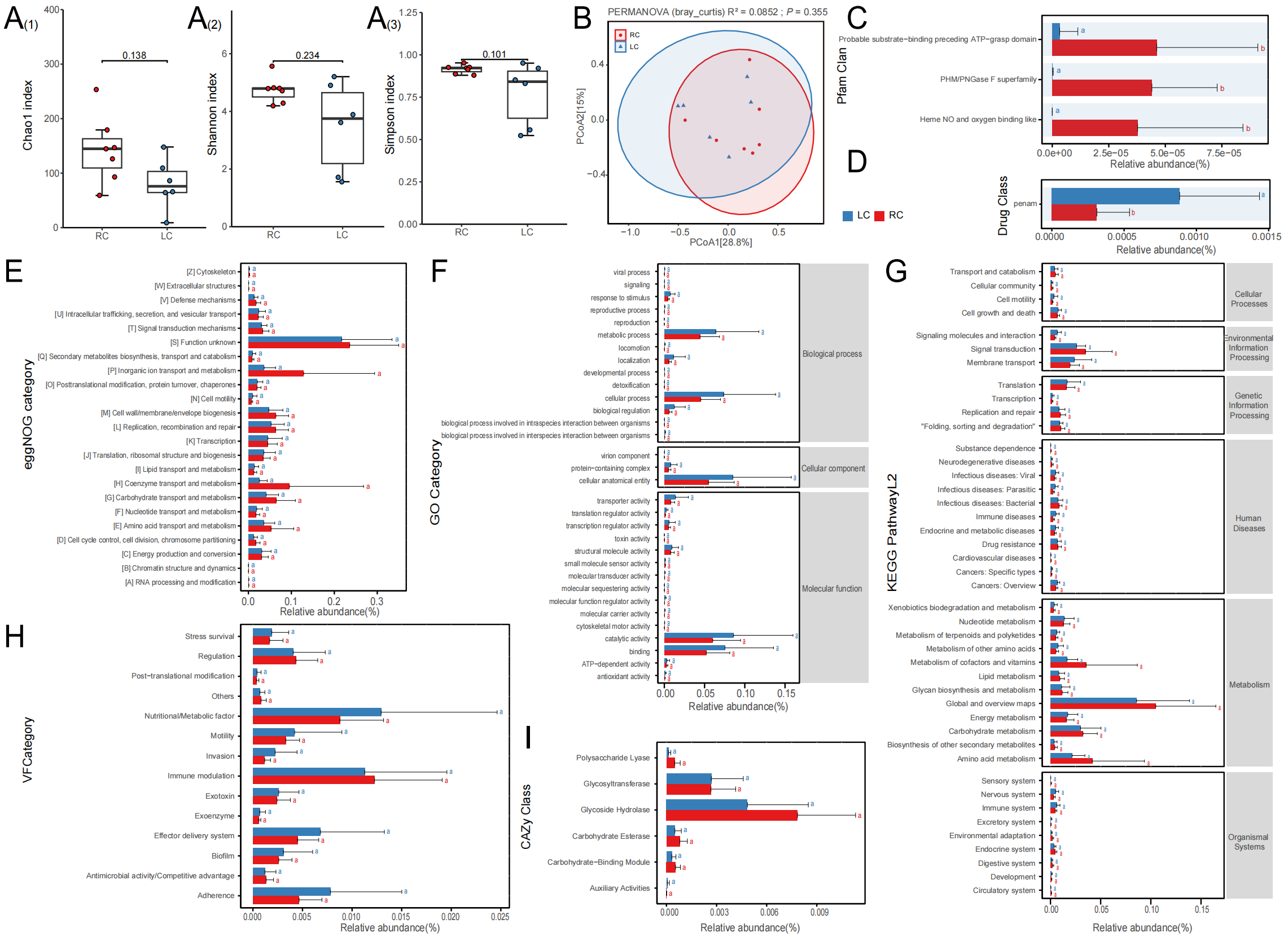

Supplement: Figure S4 — Differences in microbiomes of left and right colon cancerous tissues. [file msystems.00198-25-s0005.tif]

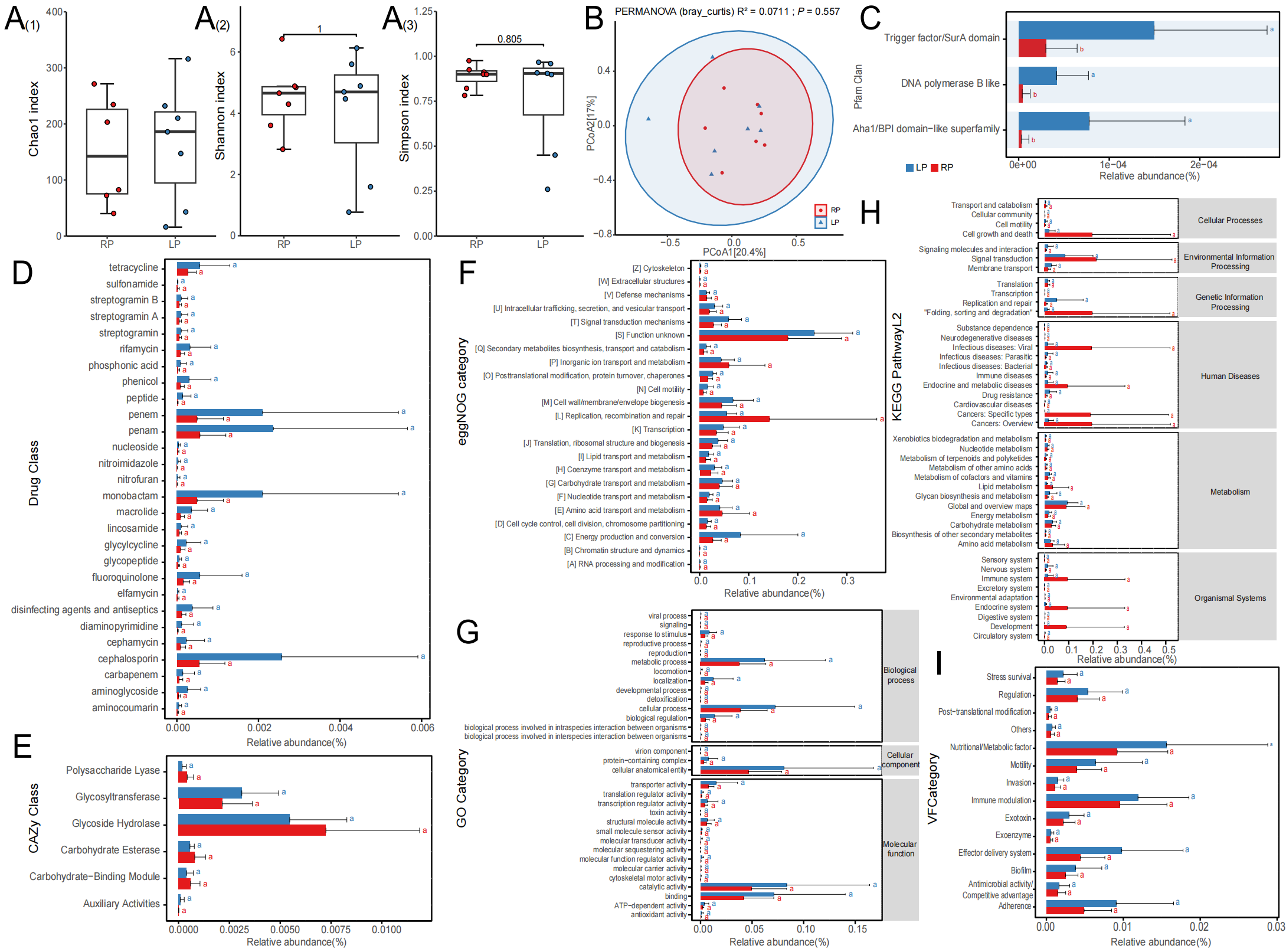

Supplement: Figure S5 — Differences in microbiomes of left and right colon paracancerous tissues. [file msystems.00198-25-s0006.tif]

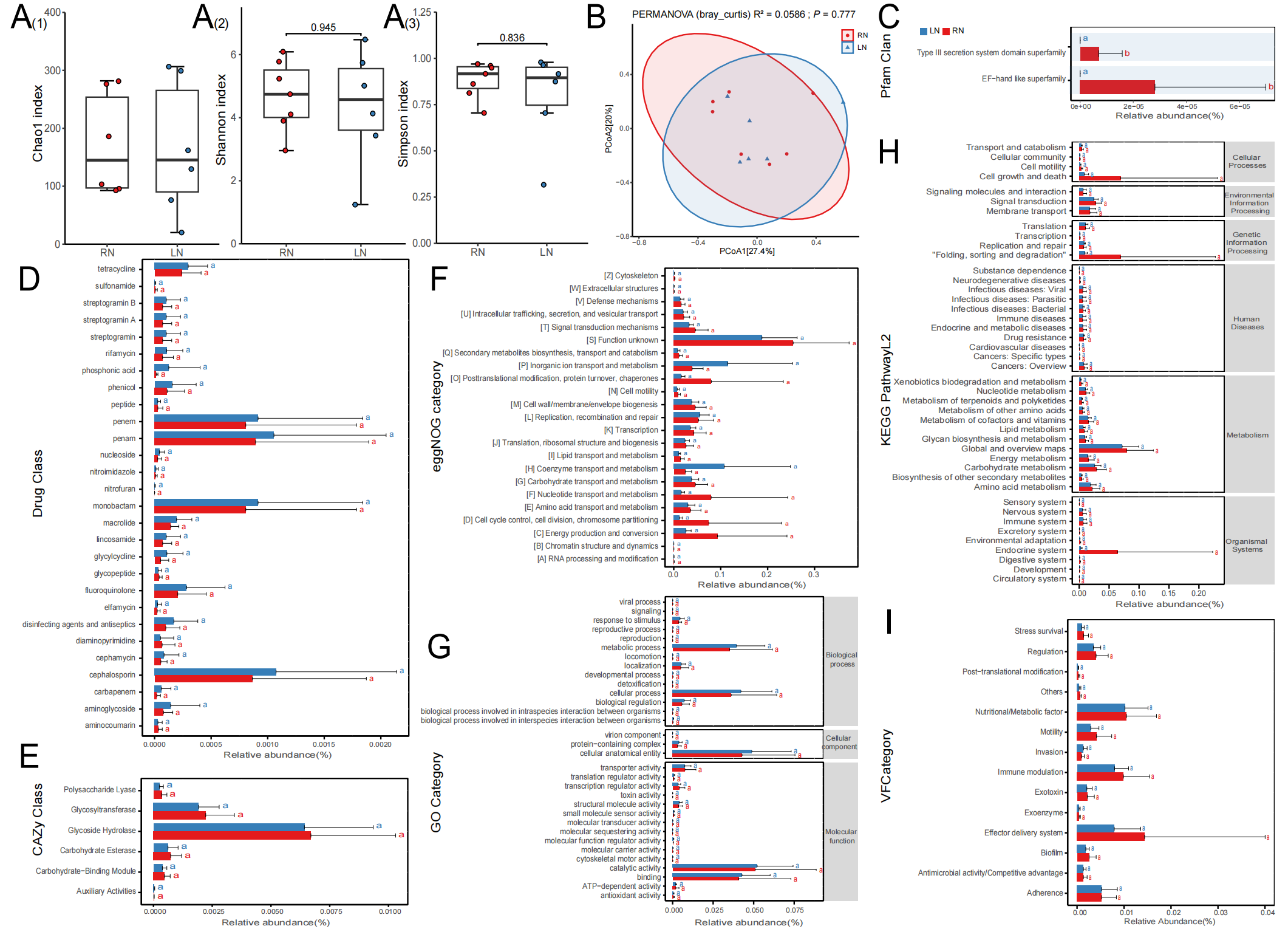

Supplement: Figure S6 — Differences in microbiomes of left and right colon normal tissues. [file msystems.00198-25-s0007.tif]

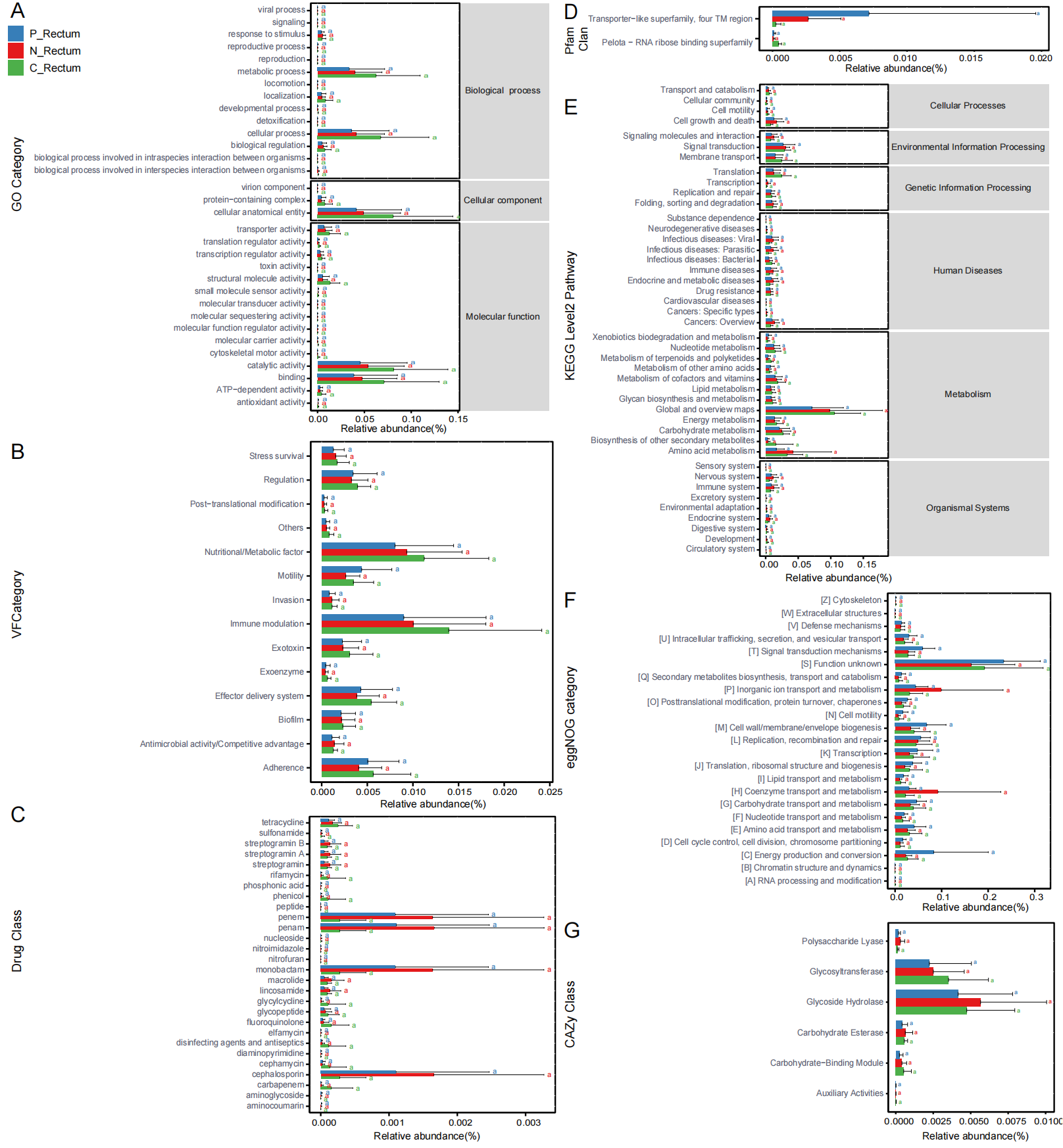

Supplement: Figure S7 — The results of the analysis based on functional pathways in the rectum. [file msystems.00198-25-s0008.tif]
