# Supplementary material for: The characteristics of tissue microbiota in different anatomical locations and different tissue types of the colorectum in patients with colorectal cancer
Source: mSystems. 2025 May 27;10(6):e00198-25. doi: 10.1128/msystems.00198-25 (PMC12172456; doi:10.1128/msystems.00198-25)
Supplement: Legends — Supplemental figure legends. [file msystems.00198-25-s0009.docx]

**Supplemental legends:**

**Figure S1. Microbial functional characteristics across different tissue types**

This figure presents the microbial functional characteristics across different tissue types (normal mucosa, peritumoral tissue, and cancerous tissue) using annotations from the KEGG Level 2 pathway (A), GO Category (B), VFCategory (C), Drug Class (D), CAZy Class (E), and eggNOG category (F) databases. Box plots are used to visually represent the functional differences among the tissue types, with distinct colors denoting separate groups.

**Figure S2. Microbial functional characteristics in the right colon**

This figure illustrates the microbial functional characteristics across various tissue types in the right colon (normal mucosa, peritumoral tissue, and cancerous tissue) using annotations from GO Category (A), KEGG Level 2 pathway (B), VFCategory (C), eggNOG category (D), Drug Class (E), and CAZy Class (F) databases. Box plots are employed to depict the functional disparities among the tissue types, with color differentiation for group distinction.

**Figure S3. Microbial functional characteristics in the left colon**

This figure delineates the microbial functional characteristics across diverse tissue types in the left colon (normal mucosa, peritumoral tissue, and cancerous tissue) using annotations from GO Category (A), KEGG Level 2 pathway (B), VFCategory (C), eggNOG category (D), Drug Class (E), and CAZy Class (F) databases. Box plots are used to visually compare the functional differences among the tissue types, with color coding for group identification.

**Figure S4. Microbial diversity and functional differences between left-sided and right-sided colon cancer tissues**

This figure examines the microbial diversity and functional differences between left-sided and right-sided colon cancer tissues. Panel A: Box plots of α-diversity indices (Chao 1 index (A1), Shannon index (A2), Simpson index (A3)) for left-sided and right-sided colon cancer tissues.

Panel B: Principal Coordinates Analysis (PCoA) based on Bray-Curtis distance, illustrating β-diversity differences between the two groups. Panels C-G: Functional analyses based on annotations from Pfam Class (C), Drug Class (D), eggNOG category (E), GO Category (F), KEGG Level 2 pathway (G), VFCategory (H), and CAZy Class (I) databases.

**Figure S5. Microbial diversity and functional differences between left-sided and right-sided colon peritumoral tissues**

This figure assesses the microbial diversity and functional differences between left-sided and right-sided colon peritumoral tissues. Panel A: Box plots of α-diversity indices (Chao 1 index (A1), Shannon index (A2), Simpson index (A3)) for left-sided and right-sided colon peritumoral tissues. Panel B: Principal Coordinates Analysis (PCoA) based on Bray-Curtis distance, highlighting β-diversity differences between the two groups. Panels C-G: Functional analyses based on annotations from Pfam Class (C), Drug Class (D), CAZy Class (E), eggNOG category (F), GO Category (G), KEGG Level 2 pathway (H), and VFCategory (I) databases.

**Figure S6. Microbial diversity and functional differences between left-sided and right-sided colon normal mucosa tissues**

This figure evaluates the microbial diversity and functional differences between left-sided and right-sided colon normal mucosa tissues. Panel A: Box plots of α-diversity indices (Chao 1 index (A1), Shannon index (A2), Simpson index (A3)) for left-sided and right-sided colon normal mucosa tissues. Panel B: Principal Coordinates Analysis (PCoA) based on Bray-Curtis distance, demonstrating β-diversity differences between the two groups. Panels C-G: Functional analyses based on annotations from Pfam Class (C), Drug Class (D), CAZy Class (E), eggNOG category (F), GO Category (G), KEGG Level 2 pathway (H), and VFCategory (I) databases.

**Figure S7. Microbial functional characteristics across different tissue types in the rectum**

This figure provides an analysis of microbial functional characteristics across different tissue types in the rectum (normal mucosa, peritumoral tissue, and cancerous tissue) using annotations from GO Category (A), VFCategory (B), Drug Class (C), Pfam Class (D), KEGG Level 2 pathway (E), eggNOG category (F), and CAZy Class (G) databases.
